# Higher doses of naloxone are needed in the synthetic opiod era

**DOI:** 10.1186/s13011-019-0195-4

**Published:** 2019-02-18

**Authors:** Ronald B. Moss, Dennis J. Carlo

**Affiliations:** Adamis Pharmaceuticals, San Diego, USA

**Keywords:** Naloxone, Fentanyl, High dose, Overdose, Opioids

## Abstract

There has been a dramatic increase of deaths due to illicit fentanyl. We examined the pharmacology of fentanyl and reviewed data on the number of repeat doses of naloxone used to treat fentanyl overdoses. Multiple sequential doses of naloxone have been required in a certain percentage of opioid overdoses due to fentanyl. In addition, fentanyl appears to differ from other opioids as having a very rapid onset with high systemic levels found in overdose victims. A rapid competition is required by naloxone to out-compete large numbers of opioid receptors occupied by fentanyl in the CNS. Taken together, we propose that higher doses of naloxone are needed to combat the new era of overdoses due to the more potent synthetic opioids such as fentanyl.

## Background

On April 5, 2018, the Surgeon General of the U. S Public Health Service took the unprecedented step of releasing a health advisory on urging the expansion of the use and access for naloxone, an opioid antagonist, to counter the ongoing opioid epidemic [1]. The Surgeon General’s recommendation was the result of a culmination of epidemiological data suggesting a sizable and rapid increase in opioid related deaths in the United States, currently averaging 115 deaths daily [1]. New data released from the Centers for Disease Control (CDC) suggests a rise of almost 10% of deaths due to drug overdoses killing approximately 72,000 Americans, a record number in 2017. The death toll is higher than the peak yearly death totals from HIV, car crashes, or gun deaths [2].

Indeed, the number of deaths due to opioids has dramatically increased over five-fold compared to 1999. Although the epidemiological data suggests that there may be rises in heroin and semisynthetic opioid use, the CDC has attributed the largest increase in deaths to illicitly manufactured synthetic opioids, such as fentanyl. This increase in opioid deaths has been described as the third wave, or the synthetic opioid era. This debate article will focus on a proposed higher dosing of naloxone in the new era of illicit potent synthetic opioids such as fentanyl.

## Main text

### The synthetic opioid era

Opioid related deaths involve both men and women of all races and adults of nearly all age groups. Although opioid abuse has been a significant public health problem in the past, the recent dramatic rise of deaths appears to be related to the abundance of synthetic opioids. This novel problem requires alternative countermeasures to deal with the significant increase in deaths including a proposed increase in naloxone dosing. The abrupt increase in synthetic opioid deaths was further substantiated by an analysis that separated synthetic opioids, such as fentanyl, into a unique category during the years of 2013–2016. [3]. Overall, the analysis revealed an 87.7% increase in deaths associated with synthetic opioids. In contrast, death rates due to natural and semisynthetic opioids remained relatively stable. Another recent report examined 64,000 drug overdoses and found that the fentanyl was responsible for more unintentional deaths than any other drug (31%) [4]. Associated with this dramatic increase in synthetic opioid abuse are studies that have suggested that current recommended doses of naloxone may be inadequate in that frequent redosing is required. [5–7]. Prior to the new synthetic opioid era, community programs reported nearly 100% naloxone post administration survival rates with current approved doses of naloxone [8]. However, the continuing rise of illicitly manufactured potent fentanyl, and even more potent analogues such as carfentanil and furantyl fentanyl, has created new challenges for the adequate treatment of overdoses. This paper examines the factors that support the use of higher doses of naloxone as a new countermeasure to the ever-changing opioid crisis.

### Pharmacology of naloxone

Naloxone is a synthetic derivative of oxymorphone that antagonizes opioids. It has been postulated to antagonize the three different opioid receptors in the brain (μ, k, and σ) [9]. The drug onset is related to its rapid entry into the brain, which is 12 to 15 times greater brain to serum ratio compared to morphine, primarily due to its high lipophilicity. The distribution half-life for naloxone has been reported to be 4.7 min while the elimination half-life averages 65 min. Naloxone competitively inhibits most opioids rapidly. Re-narconization is a phenomenon where reversal of opioid toxicity may occur but be short lived. The short duration of activity of naloxone may play a role in the re-narconization of some longer acting opioids which can result in reoccurrence of toxicity and respiratory depression. For example, in a study, 16 healthy volunteers received intravenous morphine at 0.15 mg/kg and were reversed with low dose of Naloxone (0.4 mg) [9]. Interestingly, there was a return to severe respiratory depression within 30 min. However, the issue of re-narconization may not be a major factor for shorter acting opioids such as fentanyl, as discussed later in this paper.

The reversal effects of naloxone on opioids is highly dependent not only on host factors, but also on the type of and the dose of opioid used [10].

### Host factors - metabolism and tolerance status

There are host factors that effect the metabolism of the opioids that impact on naloxone’s ability to antagonize or reverse their central nervous system manifestations. For example, the genetically polymorphic CYP2D6 is a gene that encodes for the hepatic enzyme P450 2D6. Its expression plays a role in metabolizing most opioids and has been hypothesized to play a role in interindividual response to opioids [11]. Additionally, variants in the gene encoding for μ opioid receptor may alter substrate binding or gene function impacting on opioid metabolism.

Although poorly understood, there may be different pharmacokinetic handling of naloxone in opioid dependent versus non-opioid dependent individuals. In animals, an inverse relationship was observed between physical dependence and the amount of naloxone required to elicit withdrawal jumping in mice [12]. Additionally, Fishman found that the initial plasma concentration of naloxone was 20% higher in narcotic free subjects [9]. Each opioid has different characteristics that includes μ binding affinity and the lipophilicity.

### Dose of naloxone for reversal of opioids

For naloxone, the relationship between dose of agonist and antagonist has been demonstrated in animals [13]. The dose of naloxone required for reversal compared to the dose of three opioids was compared in mice (morphine, levorphanol, and pentazocine). The higher the dose of the opioid agonist administered, the greater the dose of the naloxone needed to reverse the opioid effects on the Central Nervous System (CNS) (particularly respiratory depression).

Animal models and studies in humans support the use of higher doses of naloxone for reversal of opioid overdoses involving synthetic opioids. For example, small animal models suggest lack of clinical reversal may occur with fentanyl analogues at doses which previously reversed morphine toxicity [14]. Furthermore, animal models also suggest that the reversal of fentanyl toxicity by naloxone is dose dependent [15]. In a study of naloxone dose in beagle dogs, a 160 μg /kg i.m. naloxone regimen resulted in a nearly threefold lower odds of sedation than that of the 40 μg/ kg i.m. naloxone regimen (*P* < 0.05). Similarly, Brown et al. examined the effects of naloxone on the respiratory effects of fentanyl and alfentanil in rabbits [16]. These experiments revealed a dose dependent response for naloxone’s reversal of respiratory depression with 10 μg /kg i.m**.** resulting in greater normalization of lung minute volume due to fentanyl and alfentanil compared to with 5 μg /kg i.m**.** Similarly, the naloxone dose dependent response for reversal of fentanyl’s respiratory depression has been demonstrated in humans undergoing anesthesia [17]. The existence of naloxone resistance in animal models and dose response for reversal of fentanyl toxicity observed in animals and humans further supports the need for higher doses of naloxone not currently available.

The doses of opioids in the current era of synthetic opioids appear to be very high and clearly reaching levels which result in rapid respiratory depression and death. The high systemic levels of fentanyl found in overdoses (see Fentanyl section) supports the proposal that higher doses of naloxone are needed to adequately compete at opioid receptor binding sites.

### Opioid withdrawal syndromes with naloxone

One of the major concerns of naloxone treatment of opioid overdose is acute opioid withdrawal syndrome (OWS). The clinical effects observed in OWS due to excessive or overly rapid reversal of opioid overdose includes vomiting, seizure, delirium, and agitation [18]. OWS is more likely to occur if there is tolerance and habitual use of opioids. Although uncommon, life threatening hemodynamic adverse events thought to be due to a surge on OWS can occur, including hypertensive emergency, Acute Respiratory Distress Syndrome, ventricular tachycardia, ventricular fibrillation, and sudden death. However, there is no published information on the incidence of OWS in new synthetic opioid cases sometimes requiring multiple doses of naloxone. Thus, the risk appears to be low of OWS by increasing the doses to the levels proposed below.

### Opioid lipophilicity

Morphine has different receptor binding and lipophilicity profiles compare to fentanyl [17], but interestingly, morphine has similar receptor binding affinity (1.168 nM) to the μ receptor compared with fentanyl (1.346 nM). However, fentanyl (4.28 log P) is more lipophilic than morphine (1.07log P). In general, the analgesic onset of I.V. morphine is 6 min with a duration of 96 min. In contrast, due to its lipophilicity, fentanyl’s relative onset for I.V. administration is 2 min with a duration of action of less than 10 min [19]. The greater lipophilicity and potency of fentanyl will be discussed later, suggesting that it is a significant factor in supporting the need for higher doses of naloxone in treating fentanyl overdoses.

### Pharmacology of fentanyl

Fentanyl was first synthesized in 1960 by the Belgian company Janssen Pharmaceuticals in a search for an effective rapid-acting analgesic with high potency [20]. At the time, fentanyl was 100 to 200 times more potent than morphine in animal models. In addition, fentanyl had the fastest onset of action, and the highest therapeutic index [21]. Morphine is usually thought of as a long acting analgesic while fentanyl is considered short acting. Because morphine is not very lipid soluble, it takes a longer period of time to cross into the cellular lipid barrier of the brain (blood brain barrier). Thus, morphine’s onset is longer. Morphine has a longer duration of action compared to fentanyl due to the longer time exiting the central nervous system (CNS) [22]. Fentanyl and morphine have similar affinities for the mμ receptor (1.346uM and 1.168uM respectively [18] as discussed previously. The longer duration and onset of morphine has been described as “slow in slow out” in contrast to fentanyl, which has been described as “fast in fast out”. Fentanyl and heroin injections have very similar half-life’s (3–7 h) [8]. However, in the CNS heroin is converted to morphine which is less lipophilic and is retained in the CNS for a longer duration of action like morphine. Fentanyl, in contrast, is persistently lipophilic and thus has a shorter duration (30–60 min) due to its rapid exit from the CNS.

The onset of fentanyl and peak plasma levels are also dependent on the dosage and the type of delivery. Analgesia can occur within minutes of an intravenous delivery, in contrast to intranasal delivery which takes 5 to 10 min for pain relief [21]. Once again, the half-life of fentanyl is not consistent with its rapid and short duration of action due to its persistent lipophilicity in the CNS. Analgesia may be achieved with plasma levels of fentanyl as low as 0.2 ng/ml in opioid naïve patients, but higher levels are needed in opioid tolerant patients since fentanyl is metabolized by the cytochrome P450 system. Interactions can occur when used concomitantly with drugs that effect this isoenzyme system.

Fentanyl has become one of the most widely used opioids for the management of pain and is available for administration intravenously, transdermally and transmucosally [22]. Fentanyl, like morphine and other opioids, produces fatigue, sedation, nausea, vomiting, dizziness, bradycardia, respiratory depression, apnea, unconsciousness and death at higher doses due to μ opioid receptor stimulation [16]. Numerous analogues of fentanyl have been synthesized, including carfentanil and lofentanil, which are reported to be 100 times more potent than fentanyl [23].

Fentanyl is a commonly used anesthetic agent manufactured by pharmaceutical companies world-wide. A major source of illicit fentanyl in the US and Canada comes from laboratories in China [20]. Fentanyl is also manufactured illegally in Mexico and smuggled into the U.S. Illicit fentanyl is made into different forms including powder for injection, smoking, inhalation and tablets. Fentanyl can be mixed with heroin or cocaine to increase its potency [16]. It can also be mixed in tablets with oxycodone or alprazalom. Recent reported fatal systemic blood levels of fentanyl range from 0.5 to 162 ng/ml [24–26]. The reported EC50 (half maximum effective concentration) values for respiratory suppression with fentanyl have been reported to be 3.5+ 1.4 ng/ml [27]. High systemic levels of fentanyl have been documented in the synthetic opioid era as described above. The combination of high levels of fentanyl, the greater penetration into the CNS, and the rapidity of the reported overdoses, supports the hypothesis that higher doses of naloxone are needed in the new synthetic opioid era in order to out-compete the large number of opioid receptors occupied by fentanyl.

### Reversal of morphine and buprenorphine induced respiratory symptoms with naloxone

Few studies have examined the appropriate dose of naloxone after exposure to opioids. Heishman, et al., however, examined the effects of i.m. naloxone 6 h after a single i.m. injection of morphine (30 mg/70 kg) in six subjects with a history of chronic opioid use [28]. The degree of reversal of the morphine effects was dose related up to 10 mg naloxone intra-muscular for a 70 kg subject with no additional reversal at the 30 mg dose. Withdrawal symptoms were also dose related up to the 30 mg dose. However, withdrawal symptoms were not significantly different from baseline below the 10 mg dose. Similarly, Gal, et al. examined 1, 5, and 10 mg of naloxone intravenously in its ability to reverse buprenorphine intravenous induced respiratory depression in a single blind study [29]. Buprenorphine is a partial opioid receptor agonist. Complete reversal of opioid effects was noted at the 5 mg and 10 mg doses.

Morphine and buprenorphine are indeed different than fentanyl, which has greater lipophilicity.

### Multiple doses of naloxone in the synthetic opioid era

There are numerous studies suggesting that multiple sequential doses of naloxone have been needed for clinical reversal in the new synthetic opioid era. Faul, et al. looked at emergency medical providers from 2012 to 2015 using the National Emergency Service Information System and found that the percentage of patients receiving multiple sequential naloxone treatments increased from 14.5% in 2012 to 18.2% in 2015 [30]. The authors concluded that the increase in multiple dose naloxone indicates the prevalence of higher potency opioids. Klebacher, et al. examined the incidence of intranasal naloxone redosing in New Jersey Emergency Medical system from 2014 to 2016 [31]. This study found that the incidence of requiring a second dose of naloxone in response to an opioid overdose was 9%, with a 2% rate requiring a third dose. Marco, et al. looked at emergency department patients with opioid overdoses from 2016 to 2017 and found that the total number of naloxone dose was variable with a median from 4 to 8 mg doses required [32]. Most of these patients examined had a history of multiple overdoses. What is particularly interesting is that there was a significant negative correlation between dose and age with higher doses required in younger patients 20–30 years of age, suggesting the potential for access to the more potent synthetic opioids. Lastly, Sommerville, et al. reported characteristics of fentanyl overdose in Massachusetts from 2014 to 2016 [7]. This study reported 83% of patients required greater than 2 naloxone doses (usually nasal 2 mg/2 ml) in suspected fentanyl cases before a response was observed. However, for those fatal deaths, 36% had evidence of an overdose within seconds to minutes after drug use. Ninety percent were pulseless upon EMS arrival. The rapidity of drug overdoses due to fentanyl is particularly concerning and makes titrating naloxone very difficult. Although the dosing and type of naloxone administered in these studies in not clear, multiple doses of naloxone appears to be required. Perhaps the use of multiple doses of naloxone is due to the widespread misuse of fentanyl and its greater penetration into the CNS. The need for multiple doses of naloxone in fentanyl related opioid deaths may suggest that the initial reversal response with current dose of naloxone is inadequate. This is particularly true when high doses of potent synthetic opioids have been abused. Therefore, higher doses of naloxone are required as early as possible to out-compete the opioid binding sites occupied by fentanyl.

As we noted previously, with the sharp rise in deaths, the advent of new illicit synthetic opioids has created a new challenge in increasing opioid reversal success with naloxone. Re-narconization can result with some opioids requiring additional doses of naloxone due to their longer duration of action. However, fentanyl has a short duration of action and the studies documenting redosing have failed to report re-narconization. Therefore, it is conceivable that the need for redosing of naloxone is more likely due to the need for higher doses to compete with the higher doses and distribution of the fentanyl, rather than re-narconization.

Taken together, these studies suggest that higher doses of naloxone may provide a reasonable balance between therapeutic reversal effects and withdrawal symptoms in the new synthetic opioid era.

### Prospects for harm reduction with higher doses of naloxone in the new synthetic opioid era

The qualitative and quantitative characteristics of fentanyl and its analogues suggest that higher doses of naloxone are needed to combat this new era of overdoses. There are many reasons why higher doses of naloxone are needed in the current shifting epidemic with fentanyl use being a significant factor in the increasing morbidity and mortality from opioid abuse. Higher doses of naloxone should provide a public health need that is currently not fulfilled by the current doses of naloxone based on a number of supporting evidence which has been reviewed in this paper including: 1) Published data documenting the sequential use of naloxone doses often requiring 2 or more doses to treat fentanyl overdose, 2) The greater lipophilicity and high systemic levels observed in fentanyl which explains the rapid toxic effects seen with this class of opioids, 3) Animals models involving fentanyl or analogues which suggest naloxone resistance for doses that previously reversed non-fentanyl overdoses, 4) Additional animal data suggesting the reversal of fentanyl toxicity by naloxone is dose dependent, and 5) The changes in opioid use suggesting that fentanyl is now the leading causes of death compared to other types of opioids.

**Fig. 1:**
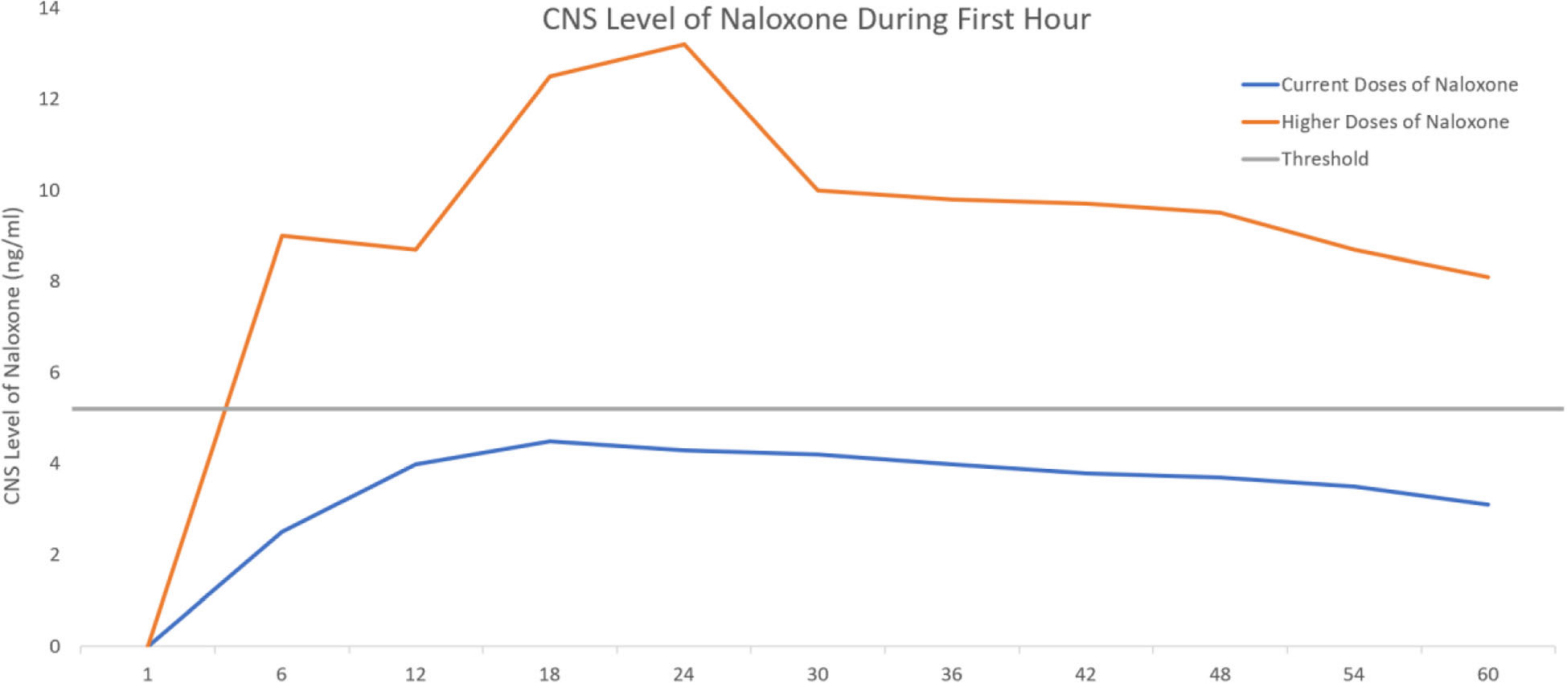
Hypothetical CNS concentrations (ng/ml) of naloxone required in the new opioid era - a threshold of naloxone may be required in the CNS during the first minutes after administration to compete with larger number of bound opiate receptors with fentanyl. This threshold may not be achieved with the current doses of naloxone, but would if higher doses of naloxone were used

Taken together, it is likely that higher doses of naloxone are needed to exceed the threshold concentrations of fentanyl at the mμ opioid receptor level in the CNS (see Fig. 1). In additional to fentanyl, more potent analogues such as carfentanil and lofentanil may require even higher doses of naloxone, as animal models suggest naloxone resistance may occur at doses which previously reversed morphine effects [33].

Currently, the highest doses of naloxone which are available for self-administration are 2 mg intramuscular (Evzio) and 4 mg intranasal product (Narcan) in the U.S. They are thought to be equivalent in terms of bioavailability [34]. Redosing is recommended as clinically indicated with no limitations and can be given every 2 to 3 min as needed [35]. It appears that in many cases of fentanyl overdoses, titrating the proper naloxone dose may not be feasible for rapid reversal and particularly for self and layperson administration where only one dose may be administered. Previously, the recommended dose of naloxone to treat opioid overdose was increased empirically by an FDA advisory committee (October 5, 2016) in the U.S. [36] as clinical studies are not feasible. After this meeting (October 19, 2016), the label dose for intramuscular naloxone increased from 0.4 mg to 2 mg [37]. This represented a five-fold increase from the previous recommended dose of naloxone.

An increased dose of naloxone can similarly be empirically determined for the new illicit more potent opioids. Based on the epidemiological data requiring sequential repeat dosing of naloxone, as well as the increased potency of fentanyl, it is reasonable to propose a range of increased doses of naloxone to 4-6 mg intramuscular or the intranasal equivalent of 8 to 12 mg. This approximates increases of 2–3 fold from the current recommended doses. This increased dose would be particularly important for layperson or self-administration where naloxone titration would not be feasible. Currently, the use of naloxone for self-administration may be limited due to access issues that may be overcome with OTC products. Because of the more rapid and potent opioids, the benefit of adequately reversing opioid toxicity outweighs the risk of opioid withdrawal syndrome. In summary, this debate paper supports a new approach that proposes to increase the dose of intramuscular naloxone to better reverse opioid intoxication, due to the unique characteristics of synthetic opioids.

## Conclusions

The concern remains that in the current opioid epidemic, without higher doses of naloxone, the risk of inadequate reversal of the opioid toxicity far exceeds the risk of over antagonizing the respiratory depression and precipitating opioid withdrawal syndrome [38]. Administering higher doses of naloxone, particularly for self or layperson administration, may be a simple countermeasure that can be initiated rapidly in an attempt to lower the morbidity and mortality in the new opioid era.

## Abbreviations

CDC: The centers for disease control
CNS: Central nervous system
OWS: Opioid withdrawal syndrome
U.S.: United States

## References

[1] https://www.surgeongeneral.gov/priorities/opioid-overdose-prevention/naloxone-advisory.html

[2] https://www.cdc.gov/drugoverdose/epidemic/index.html

[3] Seth P, Rudd RA, Noona RK, Haegerich TM. Quantifying the epidemic of prescription opioid overdose deaths. AJPH April. 2018;108(4). 10.2105/AJPH.2017.304265 PMC5844400 29513577

[4] https://www.cdc.gov/nchs/data/nvsr/nvsr67/nvsr67_09-508.pdf

[5] Schumann H, Erickson T, Thompson TM, Zautcke JL, Denton JL (2008). Fentanyl epidemic in Chicago, Illinois and surrounding Cook County. Clin Toxicol.

[6] Bell A, Bennett AS, Jones TS, Doe-Simkins M, Williams LD. Amount of naloxone used to reverse opioid overdoses outside of medical practice in a city with increasing illicitly manufactured fentanyl in illicit drug supply. Subst Abus. 2018. 10.1080/08897077.2018.1449053. 10.1080/08897077.2018.1449053 29558283

[7] Somerville NJ, O’Donnell J, Gladden RM, Zibbell JE, Green TC, Younkin M, Ruiz S, Babakhanlou-Chase H, Chan M, Callis BP, Kuramoto-Crawford J, Nields HM, Walley AY. MMWR / April 14, 2017 / Vol. 66 / No. 14. 10.15585/mmwr.mm6614a2 PMC5657806 28406883

[8] Fairbairn N, Coffin PO, Walley AY (2017). Naloxone for heroin, prescription opioid, and illicitly made fentanyl overdoses: challenges and innovations responding to a dynamic epidemic. Int J Drug Policy.

[9] Handal KA, Schauben JL, Salamone FR (1983). Naloxone. Ann Emerg Med.

[10] Sarton E, Teppema L, Dahan A (2008). Naloxone reversal of opioid induced respiratory depression with special emphasis on the partial agonist/antagonist buprenorphine. Ad Exp Med Biol.

[11] Tyndale RF, Sellers EM (2018). Opioids: the painful public health reality. Clinical Pharmacology & Therapeutics.

[12] Way EL, Loh HH (1976). Responsivity To Naloxone During Morphine Dependence. Annals NY Academy of Sciences.

[13] Dahan A, Aarts L, Smith TW (2010). Incidence, reversal, and prevention of opioid-induced respiratory depression. Anesthesiology.

[14] Wong B, Perkins MW, Tressler J, Rodriguez A, Devorak J, Sciuto AM (2017). Effects of inhaled aerosolized carfentanil on real-time physiological responses in mice: a preliminary evaluation of naloxone. Inhal Toxicol.

[15] Freise KJ, Newbound GC, Tudan C, Clark TP (2012). Naloxone reversal of an overdose of a novel, long-acting transdermal fentanyl solution in laboratory beagles. J Vet Pharmacol Ther.

[16] Brown JH, Pleuvry BJ (1981). Antagonism of the respiratory effects of Alfentanil and Fentanil by naloxone in the conscious rabbit. Br J Anaesth.

[17] Drummond GB, Davie IT, Scott DB (1977). Naloxone: dose-dependent antagonism of respiratory depression by fentanyl in anaesthetized patients. BJA: British Journal of Anaesthesia.

[18] Kim HK, Nelson LS (2015). Reducing the harm of opioid overdose with the safe use of naloxone: a pharmacologic review. Expert Opin Drug Saf.

[19] MacKenzie M, Zed PJ, Ensom MHH (2016). Opioid pharmacokinetics-pharmacodynamics: clinical implications in acute pain Management in Trauma. Annals of pharmacology.

[20] Kuczynska K, Grzonkowski P, Kacprzak L, Zawilska JB (2018). Abuse of fentanyl: an emerging problem to face. Forensic Sci Int.

[21] Stanley TH (2014). The fentanyl story, the journal of pain, Vol 15. No.

[22] Taylor DR. The pharmacology of fentanyl and its impact on the management of pain, Medscape. Neurology. 2005;7(2) https://www.medscape.org/viewarticle/518441.

[23] Burns SM, Cunningham CW, Merce, SL. DARK Classics in Chemical Neuroscience: Fentanyl, ACS Chem Neurosci. 2018;9(10):2428–2437. 10.1021/acschemneuro.8b00174. Epub 2018 Jun 12 10.1021/acschemneuro.8b00174 29894151

[24] Sutter ME, Gerona RR, Davis MT, Roche BM, Colby DK, Chenoweth JA, Adams AJ, Owen KP, Ford JB, Black HB, Albertson TE (2017). Fatal fentanyl: one pill can kill. Acad Emerg Med.

[25] Tomassoni AJ, Hawk KF, Jubanyik K, Nogee DP, Durant T, Lynch KL, Patel R, Dinh D, Ulrich A, D’Onofrio G (2017). Multiple fentanyl overdoses — New Haven, Connecticut, June 23, 2016. MMWR.

[26] Fogarty MF, Papsun DM, Logan BK. Analysis of Fentanyl and 18 Novel Fentanyl. Analogs and Metabolites by LC–MS-MS, and report of Fatalities Associated with Methoxyacetylfentanyl and Cyclopropylfentanyl. J Anal Toxicol. 2018:1–13. 10.1093/jat/bky035 29750250

[27] Lotsch J, Walter C, Parnham MJ, Oertel BG, Geisslinger G (2013). Pharmacokinetics of non-intravenous formulations of fentanyl. Clin Pharmacokinet.

[28] Heishman SJ, Stitzer ML, Bigelow GE, Liebson IA. Acute opioid physical dependence in Postaddict humans: naloxone dose effects after brief morphine exposure. J Pharmacol Exp Ther. 1989;248(1). 2913267

[29] Gal TJ (1989). Naloxone reversal of buprenorphine-induced respiratory depression. Clin Pharmacol and Ther.

[30] Faul M, Lurie P, Kinsman JM, Dailey MW, Crabaugh C, Sasser SM. Multiple naloxone administrations among emergency medical service providers is increasing. Prehospital Emergency Care. 21(4):411–9. 10.1080/10903127.2017.1315203 PMC6026856 28481656

[31] Klebacher R, Harris MI, Ariyaprakai N, Tagore A, Robbins V, Dudley LS, Bauter R, Koneru S, Hill RD, Wasserman E, Shanes A, Merlin MA (2017). Incidence of Naloxone Redosing. in the Age of the New Opioid Epidemic. Prehosp Emerg Care.

[32] Marco CA, Trautman W, Cook A, Mann D, Rasp J, Perkins O, Ballester M (2018). naloxone use among emergency department patients with opioid overdose. The Journal of Emergency Medicine.

[33] Wong B, Perkins MW, Tressler J, Rodriguez A, Devorak J, Sciuto AM. Effects of inhaled aerosolized carfentanil on real-time physiological responses in mice: a preliminary evaluation of naloxone. Inhal Toxicol. 29(2):65–74. 10.1080/08958378.2017.1282065 28330429

[34] Narcan Summary Basis of Approval, https://www.accessdata.fda.gov/drugsatfda_docs/summary_review/2017/208411s001SumR.pdf

[35] Evzio product insert. https://www.accessdata.fda.gov/drugsatfda_docs/label/2016/209862lbl.pdf

[36] FDA Advisory Committee 2016 https://www.fda.gov/downloads/AdvisoryCommittees/CommitteesMeetingMaterials/Drugs/AnestheticAndAnalgesicDrugProductsAdvisoryCommittee/UCM533421

[37] https://www.accessdata.fda.gov/drugsatfda_docs/nda/2016/209862Orig1s000SumR.pdf

[38] Lynn RR, Galinkin JL (2018). Naloxone dosage for opioid reversal: current evidence and clinical implications. Ther Adv Drug Saf.

